# Supplementary material for: Evaluation of a novel real-time adaptive assist-as-needed controller for robot-assisted upper extremity rehabilitation following stroke
Source: PLoS One. 2023 Oct 11;18(10):e0292627. doi: 10.1371/journal.pone.0292627 (PMC10566685; doi:10.1371/journal.pone.0292627)
Supplement: S1 Checklist — (DOCX) [file pone.0292627.s001.docx]

STROBE Statement—checklist of items that should be included in reports of observational studies

|  | | Item No. | | Recommendation | Page  No. | | Relevant text from manuscript | |  |  |
| --- | --- | --- | --- | --- | --- | --- | --- | --- | --- | --- |
| **Title and abstract** | | 1 | | (*a*) Indicate the study’s design with a commonly used term in the title or the abstract | 2 | | This is a pilot clinical study that investigates the use of an electromyography-based adaptive assist-as-needed controller to avoid slacking behavior during robotic rehabilitation for people with stroke. | |  |  |
|  |  |  |  | (*b*) Provide in the abstract an informative and balanced summary of what was done and what was found | 2 | | The study involved a convenience sample of five individuals with chronic stroke who underwent a robot therapy program utilizing horizontal arm tasks. The Fugl-Meyer assessment (FMA) was used to document motor impairment status at baseline. Velocity, time, and position were quantified as performance parameters during the training. Arm and shoulder surface electromyography (EMG) and electroencephalography (EEG) were used to assess the controller's performance. The cross-sectional results showed strong second-order relationships between FMA score and outcome measures, where performance metrics (path length and accuracy) were sensitive to change in participants with lower functional status. In comparison, speed, EMG and EEG metrics were more sensitive to change in participants with higher functional status. EEG signal amplitude increased when the robot suggested that the robot was inducing a challenge during the training tasks. This study highlights the importance of multi-sensor integration to monitor and improve upper-extremity robotic therapy. | |  |  |
| Introduction | | | | | |  | |  |  |  |
| Background/rationale | | 2 | | Explain the scientific background and rationale for the investigation being reported | 3 | | Several studies have shown how the strategy of control used in robot-assisted rehabilitation can significantly and positively impact the rehabilitation process's effectiveness and efficiency [3,4,6,8-13], but also highlight drawbacks such as "slacking" where the subject lets the robot drive their movements without performing sufficient physical effort to benefit from the training | |  |  |
| Objectives | | 3 | | State specific objectives, including any prespecified hypotheses | 4 | | The present study aimed to develop and validate a novel adaptive AAN (aAAN) algorithm for upper-extremity robot-assisted therapy for stroke. This new algorithm's control system integrates EMG and a haptic system to provide a subject-cooperative non-slacking robotic-assisted therapy to facilitate and accelerate upper extremity motor function recovery. Changes in performance metrics, EMG entropy amplitude, and EEG power density between the baseline and training sessions were examined for each subject. Relationships of these responses to participants' functional status using the Fugl-Meyer (FMA) clinical assessment at baseline were explored to quantify the robot training system's sensitivity. This approach enabled us to validate how outcome measures would be expected to change with improvement in functional status. | |  |  |
| Methods | | | | | |  | |  |  |  |
| Study design | | 4 | | Present key elements of study design early in the paper | 5-10 | | The following essential requirements were prescribed to develop robot-assisted training with an adaptive assist-as-needed system.” | |  |  |
| Setting | | 5 | | Describe the setting, locations, and relevant dates, including periods of recruitment, exposure, follow-up, and data collection | 9-11 | | The study setting was a university research laboratory. …. Study recruitment began in January 2020 and ended in mid-March 2020 due to the COVID pandemic shutdown. | |  |  |
| Participants | | 6 | | (*a*) *Cohort study*—Give the eligibility criteria, and the sources and methods of selection of participants. Describe methods of follow-up  *Case-control study*—Give the eligibility criteria, and the sources and methods of case ascertainment and control selection. Give the rationale for the choice of cases and controls  *Cross-sectional study*—Give the eligibility criteria, and the sources and methods of selection of participants | 9-10 | | Participants were community-dwelling adults recruited through the local rehabilitation hospital and stroke rehabilitation unit of the local primary care hospital. Individuals were first approached by their therapist (not involved in the study) and provided with contact information if they were interested in participating.  Inclusion criteria were: >19 years of age; chronic hemiplegic stroke affecting upper extremity >6 months; able to actively move their shoulder, elbow, and wrist; Modified Ashworth Scale 3 or less for all joints.  We excluded those who had: a history of severe neurological injuries other than or stroke; severe concurrent medical diseases; infections, circulatory, heart or lung, pressure sores; problematic spasticity (e.g., Modified Ashworth >3); severely compromised arm function (Fugl-Myer score < 21 on the upper-extremity scale); heterotopic ossification; significant contractures; psychiatric or cognitive situations that may interfere with the proper operation of the device; cognitive impairments resulting in an inability to follow directions; poor skin integrity in areas in contact with the device; uncontrolled Autonomic Dysreflexia; and functional visual field or hemispherical neglect. These exclusion criteria were selected based on previous research studies of robot-assisted therapy in a clinical population with impaired arms [6,20,22]. | |  |  |
|  |  |  |  | (*b*) *Cohort study*—For matched studies, give matching criteria and number of exposed and unexposed  *Case-control study*—For matched studies, give matching criteria and the number of controls per case |  | | N/A | |  |  |
| Variables | | 7 | | Clearly define all outcomes, exposures, predictors, potential confounders, and effect modifiers. Give diagnostic criteria, if applicable | 10 | | Prior to data collection, FMA scores were acquired. The next step was to set up the EEG and EMG electrodes. Nine EEG electrodes and six EMG electrodes were placed on the subject according to Fig 3. | |  |  |
| Data sources/ measurement | | 8* | | For each variable of interest, give sources of data and details of methods of assessment (measurement). Describe comparability of assessment methods if there is more than one group | 10-12 | | Six tests involving a planar 2 degree of freedom (DOF) reach task were performed: one for a baseline and five for the training. The data collection took between 45 minutes to one hour. In the first protocol, called baseline, which was used to acquire baseline characteristics, the subject was asked to reach the letters appearing on the monitor. The letters appeared in alphabetical order, one at a time, three times. First, only the first line of letters, A to E, shows up on the screen. Initially, we evaluated simple horizontal tasks with the elbow bent at this point. Next, the second line of letters, F to J shows up, again to evaluate horizontal tasks but increasing the elbow extension. Finally, the last line of letters again shows up to evaluate horizontal tasks and increase elbow extension. The letter positions can be seen in Fig 3.  After that, letters appeared in a sequence that the subject had to reach. For example, letter A shows first, then letter F, so the subject performs a reaching forward task with the elbow half extended. Next, letter B appears, then letter H requiring lateral reaching with elbow half extended, and so on. The participant is given 5 seconds to reach the target, if they are unable to get to the target within this time, the target disappears, and the next target shows up on the screen.  The baseline phase was used to collect ROM, velocity, force, and electromyography signal (EMG) information. No assistance was provided from the robot during the baseline phase.  During subsequent training protocols, the subject had to repeat the same movements as the baseline phase for the following training protocols. However, this time, the letters appeared on the screen randomly. The letters appear 30 times for training tests one, two, and three, and 60 times for training tests four and five, with an interval 5 seconds between them. The subjects had 60 seconds to rest between the tests.  In this part of the training the controller (as described above) dictates the protocol: The robot-arm assisted the participants when needed; if the subject could not reach the target, the robot-assisted the subject in getting to the target. The robot continuously evaluated the participant's EMG signal throughout the trajectory to achieve the target and only assisted if the EMG signal was still detectable and there was no movement. The robot also applied some resistance to challenge the participant if both the velocity and the EMG were higher than their respective thresholds. The EMG signal threshold necessary for the robot to assist/resist increased based on the participant's improvement. | |  |  |
| Bias | | 9 | | Describe any efforts to address potential sources of bias | 9 | | To reduce bias, inclusion criteria were broad as possible: >19 years of age; chronic hemiplegic stroke affecting upper extremity, >6 months; able to actively move their shoulder, elbow, and wrist; Modified Ashworth Scale 3 or less for all joints. | |  |  |
| Study size | | 10 | | Explain how the study size was arrived at | 9 | | The original planned design was a prospective cohort study of 10 stroke participants over 20 training sessions. Study recruitment began in February 2020 and ended in mid-March 2020 due to the COVID pandemic shutdown. As such this study focuses on the baseline training sessions of the five participants recruited and tested prior to the national lockdown. | |  |  |
| Quantitative variables | 11 | | Explain how quantitative variables were handled in the analyses. If applicable, describe which groupings were chosen and why | | 12 | | This study collected EEG, EMG, and performance data in order to analyze the effectiveness of the novel aAAN algorithm. The EMG signal was used as an input for the control system and used to assess the training effects in conjunction with the performance parameters and EEG data. | | |  |
| Statistical methods | 12 | | (*a*) Describe all statistical methods, including those used to control for confounding | | 13 | | Sensitivity of the system's responses was evaluated by using curve fitting methods to assess the relationship between the participant's FMA scale and performance metrics (average velocity, parameter $P_{1}$ and $P_{2}$), EMG entropy, and EEG power density. Details of the analyses follow. | | | |
|  |  |  | (*b*) Describe any methods used to examine subgroups and interactions | |  | | N/A | | | |
|  |  |  | (*c*) Explain how missing data were addressed | |  | | N/A | | | |
|  |  |  | (*d*) *Cohort study*—If applicable, explain how loss to follow-up was addressed  *Case-control study*—If applicable, explain how matching of cases and controls was addressed  *Cross-sectional study*—If applicable, describe analytical methods taking account of sampling strategy | |  | | N/A | | | |
|  |  |  | (*e*) Describe any sensitivity analyses | | 13 | | Finally, curve fitting was used to quantify the relationship between baseline functional status (via the FMA score) and training outcomes measures: performance metrics, EMG, and EEG. Polynomials and rationales were used to find the simplest equation that best fit the experimental data. These analyses will provide internal validity whereby performance parameters, muscle and brain signals, are expected to vary with the user's functional status, thereby revealing the system's sensitivity to modify these outcomes in future prospective interventions. | | | |
| Results | | | | | | | | | | |
| Participants | 13* | | (a) Report numbers of individuals at each stage of study—eg numbers potentially eligible, examined for eligibility, confirmed eligible, included in the study, completing follow-up, and analysed | | 13 | | Five chronic post-stroke participants were enrolled in this pilot clinical study. | | | |
|  |  |  | (b) Give reasons for non-participation at each stage | |  | | N/A | | | |
|  |  |  | (c) Consider use of a flow diagram | |  | | N/A | | | |
| Descriptive data | 14* | | (a) Give characteristics of study participants (eg demographic, clinical, social) and information on exposures and potential confounders | | 13 | | Table2: Subject demographics, clinical scores, and unadjusted performance scores. | | | |
|  |  |  | (b) Indicate number of participants with missing data for each variable of interest | |  | | N/A | | | |
|  |  |  | (c) *Cohort study*—Summarise follow-up time (eg, average and total amount) | |  | | N/A | | | |
| Outcome data | 15* | | *Cohort study*—Report numbers of outcome events or summary measures over time | |  | | N/A | | | |
|  |  |  | *Case-control study—*Report numbers in each exposure category, or summary measures of exposure | |  | | N/A | | | |
|  |  |  | *Cross-sectional study—*Report numbers of outcome events or summary measures | | 10 | | Six tests involving a planar 2 degree of freedom (DOF) reach task were performed: one for a baseline and five for the training. | | | |
| Main results | 16 | | (*a*) Give unadjusted estimates and, if applicable, confounder-adjusted estimates and their precision (eg, 95% confidence interval). Make clear which confounders were adjusted for and why they were included | | 13 | | Table2: Subject demographics, clinical scores, and unadjusted performance scores. | | | |
|  |  |  | (*b*) Report category boundaries when continuous variables were categorized | |  | | N/A | | | |
|  |  |  | (*c*) If relevant, consider translating estimates of relative risk into absolute risk for a meaningful time period | |  | | N/A | | | |

Continued on next page

| Other analyses | 17 | Report other analyses done—eg analyses of subgroups and interactions, and sensitivity analyses |  | N/A |
| --- | --- | --- | --- | --- |
| Discussion | | | | |
| Key results | 18 | Summarise key results with reference to study objectives | 18-19 | Results showed us that we achieved the aim of avoiding slacking by always providing a challenge.  Our results showed that slacking never occurred (see Fig 1) when the robot was assisting, which was accomplished by the algorithm adding sufficient challenge when performance was good.  Another relevant finding was that higher brain activity was observed during training for some subjects (Fig 6E), which is expected when more effort is required from the participant [17, 18, 21].  The results showed a strong relationship between functional status and measurement outcomes, but the relationship was not a simple straight-line linear relationship; rather, it was always a higher-order equation that best fit the data, as seen in Fig 6. The curves' shapes were particularly revealing in terms of what might be expected as functional status improves with continued training.  Performance parameters P_1norm_ (Fig. 6A) and P_2norm_ (Fig. 6B) were more sensitive to change in participants with lower FMA scores, suggesting that these parameters may be more important to monitor subjects with more severe limitations at an earlier stage of motor recovery.  Conversely, the results for velocity (Fig. 6C), mean EMG entropy (Fig. 6D), and EEG spectral density (Fig. 6E) showed that these outcomes were more sensitive to change in participants with higher functional status. Also, as reported by Tang et al. [27], entropy is a suitable feature for correlating differences in neuromuscular changes in impairments after stroke. |
| Limitations | 19 | Discuss limitations of the study, taking into account sources of potential bias or imprecision. Discuss both direction and magnitude of any potential bias | 19 | There were limitations to the study. The sample size was small, but consistent with many previous studies evaluating robot-assisted therapy's performance [23-26]. The COVID pandemic shutdown prevented a larger sample from being recruited and eliminated the possibility of longitudinal assessments. Study participants were 80% male; a larger study would be needed to have better representation from women with chronic stroke. |
| Interpretation | 20 | Give a cautious overall interpretation of results considering objectives, limitations, multiplicity of analyses, results from similar studies, and other relevant evidence | 18-19 | Results showed us that we achieved the aim of avoiding slacking by providing a challenge at all times, which can be seen in Fig 5 where the muscle activity increased during training (robot on) when compared to the baseline (robot off) even for the subject with a low score, showing that the slacking behavior is not happening even when the robot is assisting the subject as expected. Such results contradict the results presented by Dipietro et al. [16], where the EMG signal was also used to trigger the robot, and their results showed that when the robot was on, the EMG amplitude reduced for all subjects. The authors explain this decrease in EMG as a validation that their robot was assisting the subject in completing the task, but it also indicates that slacking might occur. In contrast, our results showed that slacking never occurred (see Fig 1) when the robot was assisting, which was accomplished by the algorithm adding sufficient challenge when performance was good.  Another relevant finding was that higher brain activity was observed during training for some subjects (Fig 6E), which is expected when more effort is required from the participant [17, 18, 21]. Although there was only a small change for some subjects, resulting in a statistically insignificant difference, the trend was increasing. This result suggest that the participant had to do more work when the robot was on, which may infer that the robot's use increases the engagement needed from the subject, potentially improving neuroplasticity in the long term.  The results showed a strong relationship between functional status and measurement outcomes, but the relationship was not a simple straight-line linear relationship; rather, it was always a higher-order equation that best fit the data, as seen in Fig 6. The curves' shapes were particularly revealing in terms of what might be expected as functional status improves with continued training.  Performance parameters P_1norm_ (Fig. 6A) and P_2norm_ (Fig. 6B) were more sensitive to change in participants with lower FMA scores, suggesting that these parameters may be more important to monitor subjects with more severe limitations at an earlier stage of motor recovery.  Conversely, the results for velocity (Fig. 6C), mean EMG entropy (Fig. 6D), and EEG spectral density (Fig. 6E) showed that these outcomes were more sensitive to change in participants with higher functional status. Also, as reported by Tang et al. [27], entropy is a suitable feature for correlating differences in neuromuscular changes in impairments after stroke. |
| Generalisability | 21 | Discuss the generalisability (external validity) of the study results | 3 and 19 | This study highlights the importance of multi-sensor integration as a way to monitor and improve upper-extremity robotic therapy.  Based on these findings, it may be hypothesized that participants with lower functional status may be expected to improve more in their performance measures before showing significant changes in muscle and brain activity during a longitudinal study of repeated training sessions. On the other hand, participants with higher functional status would have little performance improvement (already performing quite well). Still, they would show higher muscle and brain activity changes due to the increased challenge introduced by the aAAN algorithm for these subjects. Future studies will be needed to verify if the novel control system can accelerate motor recovery over time. |
| Other information | |  | | |
| Funding | 22 | Give the source of funding and the role of the funders for the present study and, if applicable, for the original study on which the present article is based | 1 | Coordenacao de Aperfeicoamento de Pessoal de Nivel Superior, Brasil (CAPES), #001  New Brunswick Innovation Foundation, Research Assistantship Initiative, #2019-046 |

*Give information separately for cases and controls in case-control studies and, if applicable, for exposed and unexposed groups in cohort and cross-sectional studies.

**Note:** An Explanation and Elaboration article discusses each checklist item and gives methodological background and published examples of transparent reporting. The STROBE checklist is best used in conjunction with this article (freely available on the Web sites of PLoS Medicine at http://www.plosmedicine.org/, Annals of Internal Medicine at http://www.annals.org/, and Epidemiology at http://www.epidem.com/). Information on the STROBE Initiative is available at www.strobe-statement.org.
