# Supplementary material for: Evaluation of a novel real-time adaptive assist-as-needed controller for robot-assisted upper extremity rehabilitation following stroke
Source: PLoS One. 2023 Oct 11;18(10):e0292627. doi: 10.1371/journal.pone.0292627 (PMC10566685; doi:10.1371/journal.pone.0292627)
Supplement: Attachment — Submitted filename: Review Plos 1 RAMI MOBARAK.pdf [file pone.0292627.s002.pdf]

Summary

The authors have done interesting work in suggesting an adaptive assist-as-needed method for Upper extremity rehabilitation after stroke. The flow of the suggested method is clear and reasonable. However, there were large shortcomings in the structure of the paper and the discussion and interpretation of some parts of the results as following :

Majors

- **There is a poor structure in having two sections of ''Methods'' and '' Experimental Procedure'' with several subsections within each of them making some information redundant in both sections or sparsely distributed although being related to each other. I suggest combining the 2 sections within one section called ''Materials and Methods'' with combining the closely related subsections in the current version into reduced subsections thus connecting related information and avoiding redundancy.**
- 302 Although there was no statistically significant change in EEG spectral density between 303 baseline and training (t=1.18, df=4, p=0.31), the difference trended toward increased EEG activity. **This supposed finding (trended toward increased EEG activity) is not supported by any figure or table.**

Minors

- 60 Prior studies also do 61 not incorporate adaptation of the controller based on real-time physiological performance that can 62 appropriately scale assistance or resistance to improve engagement and progression. **Needs to be referenced with some studies.**
- 59 Lacking, however, was an overall analysis of the subjects' progress, including muscle and 60 brain activity, which is crucial to monitoring if motor recovery is occurring. **Poor sentence context needs rephrasing.**
- **The hypothesis of the study** (133 If this approach is effective, we should observe subjects with higher disability at baseline 134 (lower FMA scores) improve more in movement performance between targets, and those with 135 lower disability at baseline (higher FMA) improve more in synergistic muscle and cortical activity.) **must be emphasized at the end of the introduction and not in the control algorithm subsection in the methods.**

- 176 Participants. **This subsection title does not give a clear description of its content. I suggest it to be changed into ‘’ Participants selection criteria ‘’**
- 222 The baseline phase was used to collect ROM. **The ROM (I suppose Range of motion) needs to be identified in the first use in the manuscript before directly using the abbreviation even if it’s known in the field.**
- 246 To calculate the mean adjusted EMG entropy, we first created an index based on the 247 Modified Ashworth Scale summation, **The Modified Ashworth scale summation needs to be referenced.**
- 242 relationship between the participant's FMA scale and performance metrics (average velocity, 243 parameter $P1$ and $P2$), **AND** 33 baseline. Velocity, time, and position were quantified as performance parameters during the 34 training. **AND** The performance parameters were also calculated for each move, letter 139 A to letter B being one move, B to C another move, and so on, similar to the process proposed by 140 Krebs et al. [25]. The first performance parameter is the average deviation from the trajectory. $P1$ …. **Etc : There is confusion in using the performance parameters/metrics as a general term for both velocity, time, position, P1, and P2 or for each part of them separately as being explained in a separate way in the early sections referred to above.**
- **Data availability statement is unclear and does not refer to how to access the data although it’s important for the journal policy as highlighted in the table. I suggest the authors to read the protocol of the data availability statement as listed by the journal.**
- **There was little usage of the word ‘’ we ‘’ in the manuscript, in contrast to the major rest of the paper which is in the passive form. This should by unified.**
- 343 Benitez et al. [4]. Although our sample size was small, based on these findings, we are confident 344 the post-stroke sample we recruited represented a sufficiently broad range to evaluate the behavior 345 of the new aAAN algorithm. **If there is a previous study that confirms the reliability of the evaluation of such methods for few subjects then it must be cited here, otherwise in science and from a statistical point of view there is no reason for the confidence of the results in a large population when it’s performed on a small sample even if it aligns well with the previous studies cohort size. In fact, the authors already suggested to fix this limitation in future studies.**

- **In Figure 6, The velocity which is recognized by authors as a performance measure showed a different behavior from P1,P2 which are also performance measures while similar behavior to EEG and EMG. Although P1, P2, EEG, and EMG behavior with FMA was well explained by the authors in the discussion part but they did not provide a reasonable discussion statement about the velocity pattern.**
