# Supplementary material for: Evaluation of a novel real-time adaptive assist-as-needed controller for robot-assisted upper extremity rehabilitation following stroke
Source: PLoS One. 2023 Oct 11;18(10):e0292627. doi: 10.1371/journal.pone.0292627 (PMC10566685; doi:10.1371/journal.pone.0292627)
Supplement: Attachment — Submitted filename: Review 2nd response.pdf [file pone.0292627.s004.pdf]

I’d like to thank the authors for their appropriate modifications and clarifications making the context more clear and eliminating the previous confusing points. I would accept the paper to be published only after completing this final minor point :

- Although previously highlighted and the authors said that they managed to solve this point, there's still a lot of usage of ''we'' in the context such as :

'255 drive, we normalized the EMG'

'267 baseline (robot off) and training (robot on) sessions we selected the beta wave range (16 - 24Hz)'

'334 14]. We present a novel EMG-based adaptive AAN algorithm'

'post337

stroke upper extremity rehabilitation. Is this paper we present a comprehensive cross-sectional

338 analysis to...'

'404 robot-assisted therapy performance [24-27] the sample size was small. Nevertheless, we are

405 confident the post-stroke'
