# Supplementary material for: Evaluation of a novel real-time adaptive assist-as-needed controller for robot-assisted upper extremity rehabilitation following stroke
Source: PLoS One. 2023 Oct 11;18(10):e0292627. doi: 10.1371/journal.pone.0292627 (PMC10566685; doi:10.1371/journal.pone.0292627)
Supplement: Attachment — Submitted filename: PONE-D-23-10180-R2 - Response to reviewers.docx [file pone.0292627.s005.docx]

We appreciate the prompt review of our revised manuscript and recognize the Reviewers’ time commitment to assist in further strengthening the manuscript. Below we address each remaining item raised by the Reviewer and provide the page and line numbers that correspond to the revisions in the “redline” version of the revised manuscript.

***Reviewer #1:***

*1. I’d like to thank the authors for their appropriate modifications and clarifications making the context more clear and eliminating the previous confusing points. I would accept the paper to be published only after completing this final minor point :*

*- Although previously highlighted and the authors said that they managed to solve this point, there's still a lot of usage of ''we'' in the context such as :*

We have reworded and/or rephrased the sentences below to eliminate “we/us”.

*'255 drive, we normalized the EMG'* : “…EMG entropy was normalized to an index of spasticity…” (**Pg 12 Line 254**)

*'267 baseline (robot off) and training (robot on) sessions we selected the beta wave range (16 - 24Hz)'* : “…the beta wave range (16 - 24Hz) was selected…” (**Pg 13 Line 266**)

*'334 14]. We present a novel EMG-based adaptive AAN algorithm'* : “…a novel EMG-based adaptive AAN algorithm was developed that…” (**Pg 16 Line 333**)

*'post337stroke upper extremity rehabilitation. Is this paper we present a comprehensive cross-sectional338 analysis to...'* : “A comprehensive cross-sectional analysis was conducted…” (**Pg 16 Line 336**)

*'404 robot-assisted therapy performance [24-27] the sample size was small.*

*Nevertheless, we are405 confident the post-stroke'* : “Nevertheless, participants represented a sufficiently broad range of functioning to enable observation of how people…” (**Pg 19 Line 404-6**)
